# AI-Carbon-Energy: Spillover effects and drivers in interconnected markets

**DOI:** 10.1016/j.isci.2025.114541

**Published:** 2025-12-24

**Authors:** Mingming Zhang, Yue Pan, Bin Su, Dequn Zhou

**Affiliations:** 1 College of Economics and Management, China University of Petroleum (East China), Qingdao 266580, China; 2 Energy Studies Institute, National University of Singapore, Singapore 119620, Singapore; 3 Department of Industrial Systems Engineering and Management, National University of Singapore, Singapore 117576, Singapore; 4 College of Economics and Management, Nanjing University of Aeronautics and Astronautics, Nanjing 211106, China

**Keywords:** Artificial intelligence applications, Computer modeling, Energy management

## Abstract

This study explores the spillover effects between the AI market, international carbon market, and energy markets based on a time-varying parameter vector autoregression model. Further, the study uses a multivariate quantile-to-quantile regression model to identify the macro and micro factors influencing spillover effects. The results show that there are significant spillover effects among the three markets. The AI and new energy markets are the main risk-transmitting markets with respect to the return and volatility spillovers. For skewness and kurtosis, all traditional energy markets except the gas market become risk transmitters. Across all moments, the carbon market consistently is a net recipient of risk. The spillover effects clearly vary with time. Short-term dynamics drive returns and skewness connections, and long-term effects primarily drive volatility and kurtosis connections. In addition, geopolitical risk, economic policy uncertainty, climate risk, AI technological progress, and investor attention may exert differentiated impacts on various spillover effects.

## Highlights

• Significant spillover effects exist across AI, carbon, and energy markets
• Carbon market is a consistent net recipient of risk across all moments
• Spillover effects vary clearly over time across different market dynamics
• AI technological progress generally helps to restrain spillover intensity

## Graphical abstract

**Figure:**
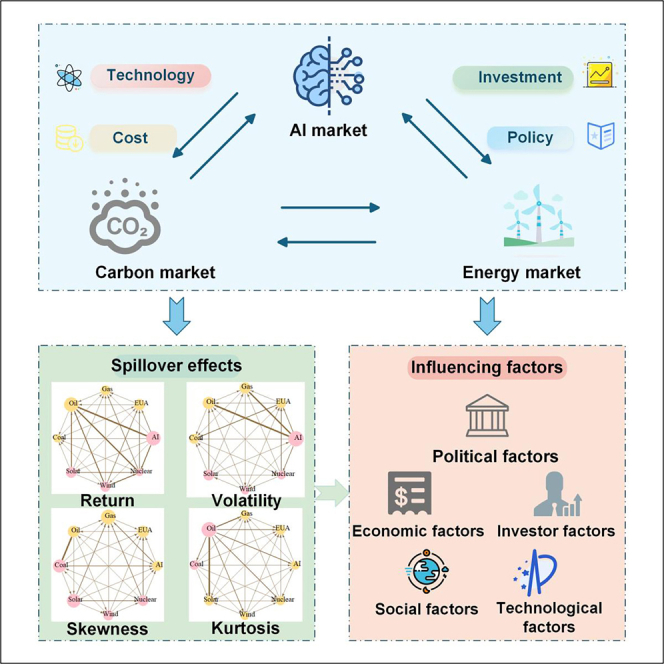

## Introduction

Artificial intelligence (AI) is broadly defined as an intelligent system capable of thinking and learning; the system includes tools, technologies, and algorithms that emulate human cognition and behavior patterns.^1^^,^^2^ AI has become a key driving force for technological innovation and industrial upgrading.^2^ AI is increasingly being integrated with energy-saving and decarbonization technologies across sectors, constructing many “AI+” industrial ecosystems. The link between the AI market and the carbon market is gradually strengthening. For example, the rapid development of AI technology, such as advances in machine learning, big data analytics, and predictive modeling, provides new tools to optimize carbon markets and forecast carbon emissions.^3^^,^^4^^,^^5^ In the development of nowadays carbon markets, AI enables in-depth analysis of trading data and policy dynamics through functions such as constructing intelligent carbon quota allocation algorithms, developing transaction risk early-warning models, and implementing dynamic assessments of emission reduction outcomes. This facilitates precise trading decision support and promotes the efficient operation of carbon markets.^6^ By 2050, the adoption of AI technologies is expected to reduce energy consumption and carbon emissions by approximately 8%–19%.^7^

Further, AI and energy markets have formed a “symbiotic relationship.” Energy is a foundational fuel for AI advancement, while AI drives cost reductions in energy consumption, increases efficiency, and optimizes resource allocation across energy systems.^8^^,^^9^ This illustrates the deep internal link among the AI, carbon, and energy markets. It is reported that 56% of global energy companies are expanding the scale of their AI projects, while 44% have integrated AI into core operational processes. AI has evolved from an optional technology to a mandatory strategic option. For instance, in the hydropower sector, China Huadian Corporation developed the world’s first large-scale runoff prediction model. Through AI algorithm optimization, it has increased hydropower utilization rates from an average of 5.8% annually over the past decade to 10.8%, demonstrating how AI significantly enhances energy efficiency.

**Figure 1:**
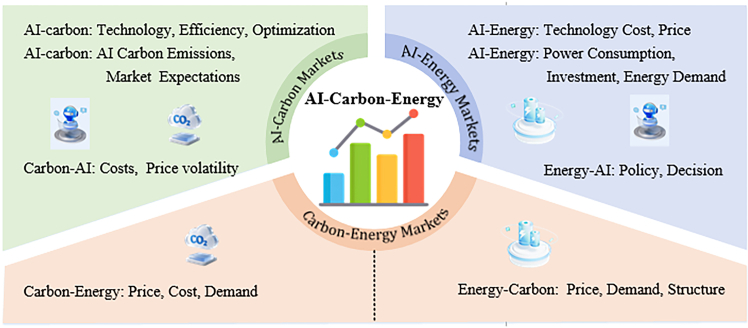
Framework of spillover effect pathways

Given the increasingly strong connections between the AI, carbon, and energy markets, the spillover effects among the three markets cannot be overlooked. Figure 1 illustrates the spillover pathways among the three markets. The risk spillover from the AI market to the carbon market primarily occurs through two pathways. First, AI can enhance production efficiency and optimize industrial structures, thereby reducing corporate costs and carbon emissions, which in turn influences carbon prices.^10^^,^^11^ Second, AI generates substantial carbon emissions. Combined with environmental pressures, this creates market expectations that the AI sector will purchase more carbon allowances, thereby influencing carbon prices.^12^^,^^13^ The spillover effects from the carbon market to the AI market can be analyzed from the perspective of corporate costs. Fluctuations in carbon prices affect the operating costs of AI enterprises, leading to stock price volatility in the AI market.

The spillover effects from the AI market to the energy market primarily manifest through two pathways, including cost pathway and investment pathway. First, AI-driven technological advances reduce the cost of new energy technologies, enhance core competitiveness, and influence energy prices. Second, the substantial energy consumption of AI computing power, compounded by environmental pressures, drives AI corporations to invest in renewable energy, thereby affecting energy demand. Then, the spillover effects from the energy market to the AI market can be analyzed from a policy perspective. For instance, when governments implement new energy subsidy policies, it may change AI corporations’ investment decisions and impact the AI market.^14^

Spillover effects also exist between carbon markets and energy markets. Carbon prices influence energy demand by affecting corporate carbon emission costs, thereby impacting energy prices.^15^^,^^16^^,^^17^ Fluctuations in energy prices affect production costs and fuel choices at the corporate level, subsequently influencing carbon emission levels and carbon allowance prices.^18^^,^^19^^,^^20^ The European Union carbon futures market is a good illustration, as it has experienced three major turbulence cycles: the 2008 financial crisis, the sovereign debt crisis, and the post-pandemic energy trilemma (COVID-19, economic rebound, and supply shocks).^21^^,^^22^ The volatility was contagious and was amplified by growing market symbiosis.

Previous studies have yielded significant theoretical and empirical findings regarding the spillover effects in markets and their influencing factors. However, there are several critical research gaps to address. AI is increasingly becoming a core technological support for carbon markets and energy markets, playing a pivotal role in carbon emission monitoring and optimizing energy structures. While the interconnection between AI and these markets continue to intensify, little scholarly attention has focused on tri-market spillover effects. Previous studies on risk transmission mechanisms predominantly rely on first- and second-order moments of asset return distributions^23^^,^^24^; these do not fully capture financial risks during extreme market conditions and do not consider higher-order moment risks. Further, previous analyses of spillover determinants tend to examine specific factors or limited combinations and have not applied a comprehensive multi-factor approach. Methodologically, studies generally focus on average effects or examine spillover quantiles in isolation, without a concurrent analysis of market spillover effects and their determinants across different quantile conditions.

This study measures the spillover effect among the AI markets, the international carbon market, the traditional energy market, and the new energy market. The research also explores the factors influencing the spillover effect. First, based on the returns, the Glosten-Jagannathan-Runkle generalized autoregressive conditional heteroskedasticity with skewness and kurtosis (GJRSK) model is used to calculate conditional volatility, skewness, and kurtosis. This introduces higher-order moments to measure asymmetric and tail risks in the markets. Second, a time-varying parameter vector autoregression (TVP-VAR) model is used to analyze static spillover effects, spillover networks, and dynamic spillover changes among carbon, energy, and AI markets. Finally, a multivariate quantile-on-quantile regression (MQQR) model reveals the influence of macro and micro factors on the spillover effects among multiple markets under different quantiles.

This is the first known study to simultaneously focus on the spillover effects among the AI, carbon, and energy markets. In doing so, it makes two key contributions in this field. First, it integrates AI market into the risk spillover analysis framework of carbon and energy markets. This facilitates the revelation of the networked characteristics of risk transmission among the three major systems, digital technology, energy transition, and environmental regulation, thereby providing the micro-foundation for constructing a complex risk theory in the “digital-green” era. It pioneers the analysis of higher-order connectedness (including asymmetric and tail risks) beyond traditional first/second moment approaches. This provides new insights into extreme market conditions that conventional spillover studies have not considered. Second, the study develops a comprehensive macro-micro analysis framework using MQQR modeling that simultaneously examines multiple influencing factors across different quantiles. This mitigates the limitations of previous studies that focus only on single macro-factors or average effects. These innovations deepen descriptions of dynamic market linkages and provide practical tools for investors and policymakers to better manage cross-market risk transmission and increase financial stability.

### The risk spillover between carbon, energy, and AI markets

Table 1 summarizes previous studies about spillover effects among the carbon, energy, and AI markets. These studies are classified based on five attributes: markets, dimensions, region/countries, periods, and methods.

**Table 1:** Summary of studies on spillover effects between the three markets

Study	Markets	Dimension	Country	Periods	Methods
Tiwari et al.^25^	Carbon and AI markets	Returns	Worldwide	2017–2020	Time-varying Markov switching copula model
Song et al.^26^	Carbon and fossil energy markets	Volatility	China	2014–2021	VAR, BEKK-MGARCH
Lovcha et al.^27^	Carbon, fossil energy, and electricity markets	Returns	Europe	2008–2018	SVAR
Qiao et al.^28^	Carbon, fossil energy, and electricity markets	Returns	Worldwide	2009–2022	TVP-VAR-SV
Su et al.^23^	Carbon and energy markets	Returns, volatility	Europe	2018–2022	QVAR
Ye et al.^29^	Carbon, fossil energy, and electricity markets	Returns	Worldwide	2017–2023	QVAR
Wu and Qin^30^	Carbon and new energy markets	Volatility	China	2014–2022	DCC-GARCH
Jiang et al.^24^	Carbon and energy markets	Volatility	Worldwide	2019–2023	QVAR
Chu et al.^31^	Carbon and fossil energy markets	Skewness, kurtosis	Worldwide	2012–2022	QVAR
Xu et al.^32^	Carbon and AI markets	Returns	China	2022–2023	TVP-VAR
Yousaf et al.^33^	Fossil energy, AI tools, and markets	Volatility	Worldwide	2019–2023	QVAR
Ghaemi Asl et al.^34^	Clean energy and AI markets	Returns	Worldwide	2018–2023	TVP-VAR
Liu et al.^35^	Energy and AI markets	Returns	Worldwide	2017–2023	Quantile regression
Zeng et al.^36^	Clean energy and AI markets	Volatility	Worldwide	2017–2023	QVAR, WLMC
Gubareva et al.^19^	Fossil energy, clean technology, and AI markets	Returns	Worldwide	2018–2023	Generalized quantile-on-quantile regression
Liu et al.^37^	Carbon and fossil energy markets	Returns	China	2021–2022	TVP-VAR
Xu et al.^38^	Carbon and energy markets	Returns	Worldwide	2012–2023	TVP-VAR-BK
Maneejuk et al.^39^	Carbon and energy markets	Volatility	Worldwide	2014–2023	Copula quantile regression

QVAR, Quantile Vector Auto-Regression; SVAR, Structural Vector Auto-Regression; TVP-VAR, Time-Varying Parameter Vector Auto-Regression; TVP-VAR-SV, Time-Varying Parameter Vector Auto-Regression with Stochastic Volatility; TVP-VAR-BK, Time-Varying Parameter Vector Auto-Regression with Bekaert-Harvey; BEKK-MGARCH, Bollerslev-Engle-Kraft-Kroner Multivariate Generalized Autoregressive Conditional Heteroskedasticity; DCC-GARCH, Dynamic Conditional Correlation Generalized Autoregressive Conditional Heteroskedasticity; WLMC, Wavelet Local Multiple Correlations.

Early studies on this topic focused on the spillover effects between energy markets and carbon markets.^23^^,^^24^^,^^26^^,^^27^^,^^28^^,^^29^^,^^30^^,^^31^^,^^37^^,^^38^^,^^39^^,^^40^ As the AI market has grown, however, its interaction with carbon markets has continued to attract more attention.^25^^,^^32^ Previous research found that AI can significantly affect carbon emissions. The combination of AI technology with energy-saving and carbon-reducing technologies can achieve energy-saving and carbon-reducing effects.^7^^,^^41^^,^^42^^,^^43^^,^^44^

In addition, scholars have begun to explore the connection between the AI market and energy market.^19^^,^^33^^,^^34^^,^^35^^,^^36^ AI provides advanced data analytics capabilities and machine learning algorithms and is now widely used to predict energy production, demand, and power generation.^8^^,^^45^^,^^46^ However, as AI-related technologies continue to develop, and AI links with the carbon and energy markets gradually strengthen, more research is needed about the spillover effects among the three markets.

In terms of the spillover dimension, most studies have focused on first- and second-moment spillover effects. First- and second-moment spillovers represent traditional and fundamental risk transmission mechanisms, referring to return and volatility spillovers, respectively. These focus on price direction and volatility magnitude. Higher-order moment spillovers represent deeper and more disruptive risk transmission. Third- and fourth-moment spillovers correspond to skewness and kurtosis spillovers, respectively, focusing on the asymmetry and heavy-tailed peaks of the return distribution. Most studies on risk transmission effects primarily focus on return and volatility spillover effects. This does not represent the financial risk under extreme market conditions and highlights the need for more research on the risks associated with higher-order moments. In terms of study area, most research has focused on the worldwide market. The research methods used in previous studies include vector auto-regression, quantile vector auto-regression, structural vector auto-regression, and Generalized Autoregressive Conditional Heteroskedasticity (GARCH) methods. These models, however, have problems with respect to overly subjective window selection and do not reflect dynamic changes in spillover effects.

In contrast, the TVP-VAR model provides a new time-varying perspective on spillover effects and does not require the independent setting of rolling window sizes. This means it avoids the problems of subjective window size settings and data loss. Given this benefit, this study simultaneously explores the spillover effects of the three markets (AI, carbon, energy) using the TVP-VAR model and explores the changes in spillover effects under extreme risks using the higher-order moments perspective.

### The factors influencing spillovers in carbon, energy, and AI markets

Understanding spillover effects among the three markets of interest is an important first step. The next step is describing the factors influencing those spillover effects, to help investors and policymakers with portfolio management and risk management. Some studies have explored the factors influencing spillovers. These studies are summarized in Table 2 and are classified based on six attributes: markets, influencing factors, periods, methods, and whether the study considers the quartile of spillovers and the quartile of influencing factors.

**Table 2:** Studies on factors that influence spillover effects

Study	Influencing factors	Markets	Periods	Methods	Quantile of spillovers?	Quantile of influencing factors ?
Tan et al.^47^	Economic policy uncertainty	Carbon, energy, and finance markets	2008–2018	Multivariate linear regression	–	–
Xiao and Wang^48^	Investor attention	Oil market	2004–2020	Linear regression	–	–
Tiwari et al.^25^	Economic policy uncertainty	Carbon and AI markets	2017–2020	Multivariate linear regression	–	–
Ma et al.^49^	Economic policy uncertainty	Electricity markets	2009–2020	Multivariate linear regression	–	–
Ding et al.^50^	Investor attention	Carbon and energy markets	2010–2021	Nonparametric causality-in-quantiles test	✓	–
Zhou et al.^16^	Climate risk	Carbon, energy, and metals markets	2015–2022	Quantile-on-quantile regression	✓	✓
Chen et al.^51^	Climate risk	Carbon and stock markets	2014–2022	Comparing different time periods	–	–
Wu and Liu^52^	Investor attention and climate policy uncertainty	Green finance markets	2014–2022	GARCH-MIDAS	–	–
Man et al.^53^	Investor attention and economic policy uncertainty	Carbon, energy, and finance markets	2014–2022	Quantile granger causality test	✓	–
Chen et al.^54^	Climate risk	Fossil and clean energy markets	2015–2022	Comparing different time periods	–	–
Guo et al.^55^	Climate risk	Energy markets	2016–2023	Quantile regression	✓	–
Xing et al.^56^	Climate risk	Clean energy and non-ferrous metals markets	2018–2023	GARCH-MIDAS	–	–
Dong et al.^40^	Climate risk	Carbon and energy markets	2017–2022	GJR-GARCH-MIDAS	–	–
Wang et al.^57^	Geopolitical risks	Energy futures markets	2018–2022	Random forest	–	–
Li et al.^58^	Geopolitical risks	Financial markets	2021–2023	Comparing different time periods	–	–
Huang et al.^59^	Geopolitical risks	Energy and metal markets	2011–2024	Quantile regression	✓	–
Liu et al.^37^	Geopolitical risks	Carbon and energy markets	2021–2022	Comparing different time periods	–	–
Zhang et al.^60^	Geopolitical risks	Energy and financial markets	2018–2022	Comparing different time periods	–	–

GARCH-MIDAS, Generalized Autoregressive Conditional Heteroskedasticity with Mixed Data Sampling; GJR-GARCH-MIDAS, Glosten-Jagannathan-Runkle GARCH-MIDAS.

In terms of markets, previous studies mainly focused on the energy, carbon, and financial markets.^37^^,^^47^^,^^55^^,^^58^^,^^60^ Studies have not yet focused on the factors influencing the spillover between AI and other markets. In terms of other influencing factors, on the macro side, some studies report that the intensity of spillover effects is influenced by factors such as geopolitical changes, economic policy changes, and extreme climate.^51^^,^^54^^,^^58^^,^^61^ In addition, some studies have applied a micro perspective to explore the factors of spillovers.^48^^,^^50^^,^^52^^,^^53^ However, most studies exploring the factors influencing spillover effects focus on a single or two influencing factors; few studies comprehensively analyze multiple influencing factors. Therefore, this study comprehensively analyzes the impact of the following factors on spillover effects from macro and micro perspectives: geopolitical risk (GPR); economic policy uncertainty (EPU); climate risk (CR); AI technological progress (measuring using patents [AITP]); and investor attention to AI, carbon, and energy markets (measured using the Google Search Volume Index [INVA]). At the macro level, GPR represents political factors, EPU represents economic factors, CR represents social factors, and AITP represents technological factors. At the micro level, INVA represents investor concerns.

In terms of methods, previous studies have mainly used a multivariate linear regression model, GARCH with mixed data sampling model, and quantile regression model. These methods only analyze average impacts or consider different quantiles of the spillover effect; this approach does not analyze both the market spillover effect and the influencing factors when in different quantiles. Therefore, this study adopts the MQQR model and considers situations where the independent and dependent variables are at different quantiles.

In conclusion, it is evident that previous studies have yielded significant findings regarding the spillover effects in markets and their influencing factors. However, several research issues remain to be further explored. First, existing studies lack discussion on spillover effects among the AI, carbon, and energy markets. It is essential to consider spillover effects among the three markets for risk prevention and investment decision-making. Second, there are insufficient analyses of spillover effects from extreme risks. When quantifying cross-market spillover effects, skewness and kurtosis spillovers should also be considered. Third, researches on the factors influencing spillover effects remains insufficient. Considering multiple factors influencing spillover comprehensively and analyzing scenarios across different quantiles would enhance the effectiveness of preventing inter-market risks.

## Results

### Static spillover effects

The data sources of markets and influencing factors are summarized in Tables 3 and 4. Table 5 shows the parameters estimated by the GJRSK model. Table 6 provides the descriptive statistics. Most of the markets show positive average returns during the sample period; European Union allowance futures (EUA) has the highest returns. The maximum and minimum values represent the gains and losses, respectively. The maximum losses of the market all exceed the maximum gains in absolute terms. Skewness and kurtosis statistics demonstrate non-normal return and volatility series distributions. Most variables show negative skewness over the sample period, indicating that most market return distributions are left-skewed; the exception is natural gas. The kurtosis values of all market return distributions exceed 3, indicating that the data distributions are sharper than the normal distribution with higher peaks and wider tails. This result is further supported by the Jarque-Bera statistics, indicating that the null hypothesis of normality is rejected in all series.

**Table 3:** Data for three markets

Market		Index	Abbreviation	Data source
Carbon market		European Union allowance futures	EUA	Intercontinental Exchange
Traditional markets	Coal market	Rotterdam coal futures	Coal	Intercontinental Exchange
	Oil market	Brent crude oil futures	Oil	Intercontinental Exchange
	Natural gas market	Port Henry natural gas futures	Gas	Energy Information Administration
New energy markets	Solar market	NASDAQ OMX Solar Index	Solar	NASDAQ website
	Wind market	NASDAQ OMX Wind Index	Wind	NASDAQ website
	Nuclear market	MVIS Global Uranium and Nuclear Index	Nuclear	MarketVector website
AI market		NASDAQ Global CTA Artificial Intelligence and Robotics Index	AI	NASDAQ website

**Table 4:** Data for influencing factors

Influencing factors	Index	Abbreviation	Data source
Political factors	Geopolitical risk index	GPR	Caldara and Iacoviello (2022)
Economic factors	Global economic policy uncertainty index	GEPU	Baker et al. (2016)
Social factors	Global frequency of climate-related disasters	CR	Emergency Events Database
Technological factors	AI patents	AITP	World Intellectual Property Organization
Investor factors	Investor attention index	INVA	Google Trends

**Table 5:** Parameters estimated by GJRSK model

Parameter		AI	EUA	Gas	Oil	Coal	Solar	Wind	Nuclear
Mean equation	*α*_1_	0.158∗∗∗	−0.040∗∗	−0.022	0.069∗∗∗	0.096∗∗∗	0.106∗∗∗	0.078∗∗∗	0.001
Variance equation	*β*_0_	0.056∗∗∗	0.363∗∗∗	0.034∗∗	0.182∗∗∗	0.075∗∗∗	0.110∗∗∗	0.046∗∗∗	0.033∗
	*β*_1_	0.036∗	0.055∗∗∗	0.039∗∗	0.027	0.025	0.039∗∗	0.056∗∗∗	0.045∗∗
	*β*_2_	0.062∗∗∗	0.018	0.004	0.099∗∗∗	0.001	0.040∗∗	0.053∗∗∗	0.035∗
	*β*_3_	0.896∗∗∗	0.878	0.956∗∗∗	0.874∗∗∗	0.969∗∗∗	0.914∗∗∗	0.877∗∗∗	0.900∗∗∗
Skewness equation	*γ*_0_	−0.147∗∗∗	−0.041∗∗	0.059∗∗∗	−0.211∗∗∗	−0.042∗∗	0.059∗∗∗	−0.048∗∗∗	−0.047∗∗
	*γ*_1_	0.029	0.012	0.019	0.192∗∗∗	0.147∗∗∗	0.004	0.006	−0.088∗∗∗
	*γ*_2_	−0.050∗∗∗	−0.002	0.021	−0.188∗∗∗	−0.149∗∗∗	0.052∗∗∗	0.088∗∗∗	0.196∗∗∗
	*γ*_3_	0.186∗∗∗	0.007	0.363∗∗∗	0.175∗∗∗	−0.002	0.218∗∗∗	−0.102∗∗∗	−0.109∗∗∗
Kurtosis equation	*δ*_0_	1.492∗∗∗	2.928∗∗∗	1.432∗∗∗	1.803∗∗∗	3.504∗∗∗	1.653∗∗∗	2.616∗∗∗	3.258∗∗∗
	*δ*_1_	0.007	0.009	0.000	0.000	0.000	0.000	0.007	0.000
	*δ*_2_	0.049∗∗∗	0.013	0.023	0.000	0.000	0.015	0.000	0.053∗∗∗
	*δ*_3_	0.521∗∗∗	0.125∗∗∗	0.581∗∗∗	0.460∗∗∗	0.239∗∗∗	0.503∗∗∗	0.220∗∗∗	0.000

^∗∗∗^, ^∗∗^, and ^∗^ denote 1%, 5%, and 10% level of significance, respectively.

**Table 6:** Descriptive statistics

	Min	Max	Mean	Median	Skewness	Kurtosis	Std. Dev	J-B	ADF	Ljung-Box (10)	ARCH-LM (10)	N
AI	−10.48	9.10	0.02	0.09	−0.49	5.73	1.38	2507.50∗∗∗	−11.21∗∗∗	894.12∗∗∗	361.45∗∗∗	1772
EUA	−17.73	16.14	0.12	0.10	−0.46	4.40	2.74	1496.20∗∗∗	−12.45∗∗∗	198.83∗∗∗	114.90∗∗∗	1772
Gas	−21.33	21.03	0.00	0.00	0.04	4.39	3.88	1425.90∗∗∗	−11.01∗∗∗	99.66∗∗∗	80.97∗∗∗	1772
Oil	−26.00	13.52	0.01	0.19	−1.43	16.21	2.41	20057.00∗∗∗	−12.21∗∗∗	247.18∗∗∗	151.53∗∗∗	1772
Coal	−53.69	32.62	0.01	0.00	−2.54	66.49	3.17	329075.00∗∗∗	−11.93∗∗∗	63.47∗∗∗	51.48∗∗∗	1772
Solar	−14.96	11.32	0.03	−0.02	−0.17	3.81	2.25	1086.10∗∗∗	−11.14∗∗∗	480.01∗∗∗	246.79∗∗∗	1772
Wind	−12.59	9.89	0.02	0.05	−0.58	11.26	1.22	9493.80∗∗∗	−11.39∗∗∗	912.18∗∗∗	388.06∗∗∗	1772
Nuclear	−10.85	7.35	0.03	0.06	−0.84	12.49	1.13	11761.00∗∗∗	−12.44∗∗∗	1414.00∗∗∗	552.64∗∗∗	1772

J-B, Jarque-Bera test; ADF, Augmented Dickey-Fuller test; ARCH-LM, Autoregressive Conditional Heteroskedasticity-Lagrange Multiplier test.

^∗∗∗^, ^∗∗^, and ^∗^ denote 1%, 5%, and 10% level of significance, respectively.

In addition, the study performs a unit root test using the Augmented Dickey-Fuller test. The result shows that all the variables are smooth at 1% level of significance. The autoregressive conditional heteroskedasticity-Lagrange multiplier test confirms the presence of an autoregressive conditional heteroskedasticity effect in this time series. Therefore, the series characteristics make it appropriate to use the GJRSK model and the spillover analysis model.

This study uses the TVP-VAR model to analyze the static spillover effects of the carbon, energy, and AI markets. The frequency domain has two types of timescales, including high frequency (1–5 trading days) and low frequency (>5 trading days). This enables a comprehensive understanding of the entire transmission chain from “short-term information shocks” to “long-term risks” through a multi-timescale perspective. High-frequency spillovers primarily capture instantaneous linkages driven by short-term market sentiment and breaking news events. It reflects the market’s rapid reaction and digestion of immediate information, exhibiting high volatility but weak persistence. Low-frequency spillovers mainly capture long-term shocks driven by macroeconomic trends and structural policy adjustments, characterized by slow changes but stable trends. Tables 7, 8, 9, and 10 show the results of static spillovers between the carbon, energy, and AI markets. “TO” represents the value of the spillover transmitted to other markets, and “FROM” represents the value of spillovers received from other markets. “NET” represents the net spillover. A positive net spillover value indicates that the market is a net transmitter of risk, and a negative net spillover value indicates that the market is a net receiver of risk. “TCI” represents the total spillover and measures the overall extent of information transfer and spillover among markets.

**Table 7:** Return spillover results

	AI	EUA	Gas	Oil	Coal	Solar	Wind	Nuclear	FROM
**Panel A: Total spillover**									

AI	45.88	1.59	0.52	2.82	0.37	19.78	16.83	12.2	54.12
EUA	2.72	81.87	1.42	3.16	3.55	1.95	2.37	2.95	18.13
Gas	1.1	1.67	90.22	1.4	1.46	1.4	0.94	1.79	9.78
Oil	4.83	3.25	1.42	75.75	1.82	3.9	3.6	5.43	24.25
Coal	0.82	3.16	1.35	2.02	91.16	0.45	0.41	0.62	8.84
Solar	20.15	1.33	0.84	2.42	0.29	47.88	18.47	8.63	52.12
Wind	17.45	1.51	0.59	2.29	0.34	18.71	44.54	14.57	55.46
Nuclear	13.68	1.87	0.95	3.49	0.25	9.55	15.97	54.24	45.76
TO	60.76	14.37	7.09	17.61	8.08	55.75	58.6	46.19	TCI
NET	6.64	−3.76	−2.69	−6.64	−0.77	3.64	3.14	0.44	38.35

**Panel B: High frequency (1 day to 5 days)**									

AI	34.89	1.23	0.37	2.23	0.25	14.59	12.66	9.16	40.5
EUA	2.1	67.73	1.19	2.64	3.1	1.55	1.94	2.43	14.95
Gas	0.92	1.41	75.48	1.21	1.11	1.13	0.77	1.38	7.93
Oil	3.59	2.65	1	60.92	1.3	2.89	2.83	3.95	18.21
Coal	0.54	2.53	1.08	1.44	71.18	0.32	0.29	0.42	6.62
Solar	15.36	0.96	0.57	1.83	0.24	36.52	13.79	6.4	39.15
Wind	12.26	1.1	0.45	1.75	0.25	12.86	33.63	10.24	38.91
Nuclear	10.41	1.45	0.68	2.84	0.18	7.14	12.52	41.58	35.21
TO	45.18	11.33	5.34	13.94	6.43	40.48	44.79	33.99	TCI
NET	4.68	−3.62	−2.59	−4.28	−0.19	1.34	5.88	−1.22	28.78

**Panel C: Low frequency (5 days to infinity)**									

AI	10.98	0.36	0.16	0.59	0.12	5.19	4.17	3.04	13.62
EUA	0.62	14.14	0.23	0.52	0.45	0.4	0.43	0.52	3.18
Gas	0.19	0.26	14.74	0.19	0.35	0.27	0.18	0.41	1.85
Oil	1.24	0.6	0.42	14.83	0.51	1.01	0.77	1.48	6.04
Coal	0.28	0.63	0.27	0.58	19.98	0.13	0.12	0.2	2.22
Solar	4.79	0.36	0.26	0.59	0.05	11.36	4.68	2.23	12.97
Wind	5.19	0.41	0.14	0.54	0.09	5.85	10.9	4.34	16.55
Nuclear	3.28	0.42	0.26	0.65	0.07	2.42	3.45	12.67	10.55
TO	15.58	3.04	1.75	3.68	1.64	15.27	13.81	12.21	TCI
NET	1.96	−0.14	−0.1	−2.36	−0.57	2.3	−2.74	1.66	9.57

**Table 8:** Volatility spillover results

	AI	EUA	Gas	Oil	Coal	Solar	Wind	Nuclear	FROM
**Panel A: Total spillover**									

AI	40.24	4.38	2.92	5.15	2.87	16.44	14.72	13.28	59.76
EUA	9.64	60.39	3.34	3.24	2.62	6.13	7.28	7.36	39.61
Gas	10.57	4.8	61.22	5.4	5.8	5.5	2.89	3.82	38.78
Oil	14.69	5.94	2.73	48.28	2.44	10.51	6.5	8.92	51.72
Coal	4.68	6.47	6.08	2.51	66.73	5.98	4.07	3.47	33.27
Solar	15.14	4.16	2.73	6.04	3.57	38.65	18.26	11.46	61.35
Wind	15.42	3.88	3.49	6.11	3.33	16.82	33.94	17.02	66.06
Nuclear	17.61	3.32	3.26	5.77	4.72	12.88	15.1	37.35	62.65
TO	87.75	32.94	24.54	34.22	25.36	74.25	68.82	65.33	TCI
NET	27.99	−6.67	−14.24	−17.5	−7.91	12.9	2.76	2.67	59.03

**Panel B: High frequency (1 day to 5 days)**									

AI	2.59	0.09	0.09	0.1	0.11	0.61	0.47	0.48	1.94
EUA	0.22	5.14	0.23	0.22	0.15	0.19	0.22	0.21	1.44
Gas	0.2	0.07	1.85	0.05	0.11	0.08	0.09	0.12	0.72
Oil	0.35	0.32	0.18	5.8	0.09	0.31	0.28	0.38	1.9
Coal	0.07	0.08	0.11	0.05	2.02	0.05	0.08	0.07	0.51
Solar	0.73	0.18	0.1	0.15	0.05	2.65	0.63	0.37	2.24
Wind	0.72	0.13	0.19	0.21	0.09	0.91	3.35	0.9	3.13
Nuclear	0.5	0.09	0.09	0.19	0.09	0.32	0.62	2.76	1.9
TO	2.79	0.95	0.99	0.98	0.69	2.45	2.39	2.54	TCI
NET	0.86	−0.49	0.27	−0.93	0.19	0.22	−0.75	0.64	1.97

**Panel C: Low frequency (5 days to infinity)**									

AI	37.66	4.29	2.83	5.06	2.76	15.84	14.26	12.8	57.82
EUA	9.42	55.25	3.11	3.02	2.47	5.94	7.06	7.14	38.17
Gas	10.37	4.74	59.37	5.35	5.69	5.42	2.79	3.7	38.06
Oil	14.33	5.62	2.55	42.47	2.36	10.2	6.22	8.54	49.82
Coal	4.61	6.39	5.97	2.46	64.72	5.93	3.99	3.4	32.76
Solar	14.41	3.97	2.62	5.89	3.51	36	17.63	11.09	59.11
Wind	14.71	3.75	3.3	5.9	3.24	15.91	30.59	16.12	62.93
Nuclear	17.11	3.23	3.17	5.58	4.63	12.56	14.48	34.59	60.75
TO	84.95	31.99	23.55	33.25	24.66	71.8	66.43	62.79	TCI
NET	27.13	−6.18	−14.51	−16.58	−8.1	12.69	3.5	2.04	57.06

**Table 9:** Skewness spillover results

	AI	EUA	Gas	Oil	Coal	Solar	Wind	Nuclear	FROM
**Panel A: Total spillover**									

AI	74.85	1.29	0.53	0.98	1.71	8.86	6.26	5.5	25.15
EUA	2.04	86.17	2.9	0.91	1.82	2.26	1.62	2.27	13.83
Gas	1.88	2.86	86.55	0.85	1.15	2.52	1.69	2.5	13.45
Oil	0.7	1.81	0.6	89.79	5.01	0.6	0.46	1.01	10.21
Coal	0.39	0.91	0.99	2.03	92.93	0.41	0.64	1.69	7.07
Solar	7.72	2.12	1.15	0.86	0.51	71.92	11.02	4.68	28.08
Wind	5.42	0.66	0.93	0.77	0.4	12.21	70.92	8.69	29.08
Nuclear	4.94	0.91	0.91	0.41	0.82	6.14	9.8	76.07	23.93
TO	23.09	10.57	8.02	6.83	11.43	33.02	31.5	26.34	TCI
NET	−2.06	−3.26	−5.43	−3.38	4.36	4.94	2.42	2.41	21.54

**Panel B: High frequency (1 day to 5 days)**									

AI	55.42	0.99	0.37	0.6	1.3	5.19	3.64	3.41	15.52
EUA	1.54	66.98	2.34	0.69	1.26	1.54	0.99	1.59	9.95
Gas	1.23	1.59	57.29	0.5	0.66	1.62	1.02	1.68	8.29
Oil	0.38	0.93	0.29	64.66	1.95	0.37	0.33	0.57	4.83
Coal	0.2	0.52	0.69	1.37	68.42	0.23	0.43	0.55	3.99
Solar	5.33	1.49	0.72	0.51	0.27	50.12	7.29	3.03	18.64
Wind	4.12	0.54	0.67	0.59	0.26	8.66	58.89	6.86	21.69
Nuclear	3.65	0.76	0.67	0.28	0.42	4.12	6.78	62.12	16.68
TO	16.46	6.82	5.73	4.55	6.12	21.72	20.47	17.7	TCI
NET	0.94	−3.12	−2.56	−0.28	2.13	3.08	−1.22	1.03	14.23

**Panel C: Low frequency (5 days to infinity)**									

AI	19.43	0.3	0.17	0.38	0.41	3.67	2.62	2.09	9.64
EUA	0.5	19.19	0.57	0.22	0.57	0.72	0.63	0.68	3.88
Gas	0.65	1.27	29.26	0.35	0.49	0.91	0.67	0.82	5.16
Oil	0.32	0.88	0.32	25.13	3.06	0.24	0.13	0.44	5.38
Coal	0.19	0.39	0.3	0.67	24.51	0.18	0.21	1.14	3.08
Solar	2.39	0.64	0.44	0.35	0.24	21.8	3.74	1.65	9.44
Wind	1.3	0.12	0.26	0.18	0.14	3.56	12.03	1.83	7.39
Nuclear	1.29	0.15	0.24	0.13	0.4	2.02	3.02	13.95	7.25
TO	6.63	3.75	2.29	2.28	5.31	11.3	11.03	8.64	TCI
NET	−3	−0.13	−2.88	−3.1	2.23	1.86	3.64	1.39	7.32

**Table 10:** Kurtosis spillover results

	AI	EUA	Gas	Oil	Coal	Solar	Wind	Nuclear	FROM
**Panel A: Total spillover**									

AI	50.79	1	1.99	15.47	7.97	8.49	8.58	5.71	49.21
EUA	2.48	65.18	2.67	14.11	7.81	2.58	2.23	2.93	34.82
Gas	3.4	2.34	61.71	15.84	10.43	1.9	1.93	2.46	38.29
Oil	3.06	2.1	2.64	54.93	27.98	2.27	4.74	2.27	45.07
Coal	2.51	2	2.67	37.9	48.85	0.89	3.17	2.02	51.15
Solar	9.77	1.03	2.42	23.34	8.97	42.93	6.77	4.78	57.07
Wind	7.57	0.67	1.33	16.69	9.24	5.4	53.66	5.45	46.34
Nuclear	5.78	1.1	1.92	13.16	6.28	7.51	8.22	56.02	43.98
TO	34.57	10.24	15.63	136.5	78.68	29.04	35.63	25.62	TCI
NET	−14.64	−24.58	−22.66	91.43	27.53	−28.02	−10.71	−18.35	52.27

**Panel B: High frequency (1 day to 5 days)**									

AI	25.27	0.2	0.36	1.02	0.57	3.91	2.98	2.04	11.07
EUA	1.35	48.25	1.66	3.33	1.78	1.13	0.98	1.99	12.22
Gas	0.81	0.71	27.03	1.71	1.14	0.73	0.38	0.65	6.13
Oil	0.72	0.34	0.35	11.74	4.53	1.08	1.36	0.45	8.82
Coal	0.57	0.25	0.58	4.72	15.41	0.2	0.74	0.24	7.31
Solar	4.18	0.25	0.56	3.01	0.83	22.02	2.52	1.79	13.14
Wind	4.29	0.18	0.34	3.03	1.72	2.94	35	2.93	15.43
Nuclear	3.45	0.67	0.9	3.88	1.96	4.88	4.15	42.94	19.9
TO	15.37	2.59	4.75	20.71	12.54	14.86	13.12	10.08	TCI
NET	4.3	−9.62	−1.38	11.88	5.23	1.72	−2.31	−9.82	13.43

**Panel C: Low frequency (5 days to infinity)**									

AI	25.53	0.8	1.62	14.45	7.4	4.58	5.61	3.68	38.13
EUA	1.14	16.94	1.01	10.77	6.03	1.45	1.24	0.95	22.6
Gas	2.59	1.63	34.67	14.13	9.29	1.17	1.54	1.82	32.17
Oil	2.35	1.76	2.29	43.19	23.44	1.2	3.38	1.83	36.24
Coal	1.94	1.74	2.08	33.18	33.44	0.68	2.43	1.78	43.84
Solar	5.58	0.78	1.86	20.33	8.13	20.91	4.25	2.98	43.93
Wind	3.28	0.49	0.99	13.65	7.52	2.46	18.66	2.52	30.91
Nuclear	2.33	0.43	1.02	9.28	4.33	2.63	4.06	13.08	24.08
TO	19.2	7.64	10.88	115.79	66.14	14.18	22.51	15.54	TCI
NET	−18.93	−14.96	−21.28	79.55	22.3	−29.74	−8.4	−8.53	38.84

Table 7 shows the spillover effects for returns. The total return spillover effect is 38.35%. This means that, on average, 38.35% of the price movements in each market are due to price movements in other markets. The AI market (60.76%) and the wind market (58.6%) have the strongest spillover effect (TO). This highlights the dominant role of the AI and wind markets in risk transmitting. This may be because rapid growth in the AI and wind energy markets is accompanied by technological uncertainty; the associated risks can quickly spread to other markets. These are also large receiving markets (FROM), indicating that markets with high-risk spillovers are also susceptible to shocks in other markets. At high and low frequencies, the total return spillover values are 28.78% and 9.57%, respectively. The connectedness decreases as the frequency bands increase. This indicates that the return spillover effects between these markets are more affected by short-term effects. The AI market is the largest spillover market in both the short and long term, and the solar and wind markets are the largest receiving markets.

Table 8 shows the results of the volatility spillovers. The total volatility spillover is 59.03%. This indicates that, on average, 59.03% of the market volatility is due to the volatility of the other markets. This demonstrates the strong volatility spillover effect among the three markets. AI is the largest-risk spillover market (87.75%), and wind energy is the largest-risk receiving market (66.06%) for the volatility spillover. This result aligns with the results for return spillovers. Meanwhile, the oil market is a net risk receiver in both the return and volatility spillovers. This is mainly because oil plays a crucial role in the global energy supply and is highly influenced by other markets. The total systematic spillover values at high and low frequencies are 1.97% and 57.06%, respectively. In both the short and long run, the AI market is the largest-risk spillover market, and the wind energy market is the largest-risk receiving market.

Table 9 shows the results of skewness spillovers. The total skewness spillover effect is 21.54%. This indicates that 21.54% of the asymmetry in the distribution of asset returns across markets is caused by other markets. In this case, the solar market is the largest-risk spillover market for the skewness spillover (33.02%) and the wind market is the largest-risk receiving market for the skewness spillover (29.08%). The total skewness spillover values at high and low frequencies are 14.23% and 7.32%, respectively. This indicates that short-term effects have a larger influence on the skewness spillover effect between these markets. Meanwhile, risk spillovers in the markets are heterogeneous across frequency bands. The risk transmitters and receivers in the markets vary with frequency bands.^40^ Specifically, the AI market is a net risk transmitter in the short term and shifts to being a net risk receiver in the long term. This may be because, in the short term, the high initial investment and technological uncertainty associated with AI lead to risks being easily transmitted to other markets. In the long term, however, the AI technology matures, and its specific applications depend on the carbon and energy markets. As such, the AI market may receive more risk transfers.

Table 10 shows the results associated with kurtosis spillovers. The total skewness spillover effect is 52.27%. This indicates that 52.27% of the probability of extreme returns occurring in each market is caused by other markets. This shows a strong kurtosis spillover effect among the three markets. The crude oil market is the largest-risk spillover market (136.5%), and the solar market is the largest-risk receiving market for the kurtosis spillover (57.07%). The risk spillover effect of the crude oil market is significantly stronger in the higher-order moments. At high and low frequencies, the total system spillover values are 13.43% and 38.84%, respectively. This indicates that long-term effects have a greater influence on the kurtosis spillover effect between these markets. In the long term, the AI market shifts from a net risk transmitter to a receiver, consistent with the findings of skewness spillovers.

### Network connectedness analysis

**Figure 2:**
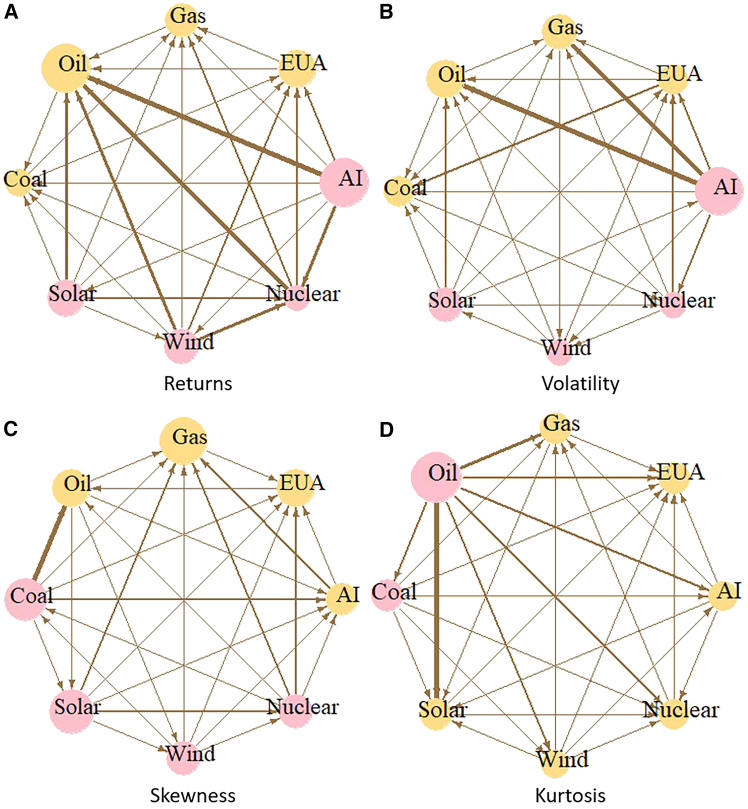
Net-pairwise directional connectedness (A) Return spillover effect, (B) Volatility spillover effect, (C) Skewness spillover effect, (D) Kurtosis spillover effect.

This section builds upon these static spillover effects to further explore the network connectedness between markets, thereby facilitating a more intuitive understanding of spillover effects across markets. Figure 2 shows the net pairwise directional connectedness of the markets. The color of each node in the graph represents the market’s role in the spillover effect: pink indicates the market is a net transmitter, and yellow indicates the market is a net receiver. The size of the node indicates the market’s net pairwise spillover value. The arrow represents the direction of the spillover. Specifically, an arrow pointing from market A to market B indicates that market A transmits risk to market B. The thickness of the lines in the network diagram indicates the intensity of spillovers between markets; thicker line segments indicate stronger linkages between the two markets.

Figure 2A shows that the AI market is the main transmitter in the return spillover network; the oil and carbon markets are the main receivers. This means the market price changes is mainly transmitted from the AI and new energy markets to the carbon and traditional energy markets. Figure 2B shows that the results of the volatility spillover network are similar to the results of the return connectedness network. This may be because AI and new energy have high investment demands and high uncertainty during development. As such, returns and volatility risks are quickly transmitted to other markets.

Figure 2C shows that, in the skewness spillover network, the AI market shifts from being a risk transmitter to being a risk receiver, and the coal market shifts to being a risk transmitter. Figure 2D shows that, in the kurtosis spillover network, all markets are risk receivers except for coal and oil markets. This indicates that the risk of extreme returns occurring is mainly transmitted from the coal and oil markets to other markets. The coal market is a net risk receiver in return spillovers and volatility spillovers. However, it transforms into a net risk transmitter in skewness and kurtosis spillovers. This indicates that the coal market rapidly propagates risk to other markets during extreme events. This highlights the need to monitor the skewness and kurtosis impacts of coal on other markets.

### Dynamic spillover effects

**Figure 3:**
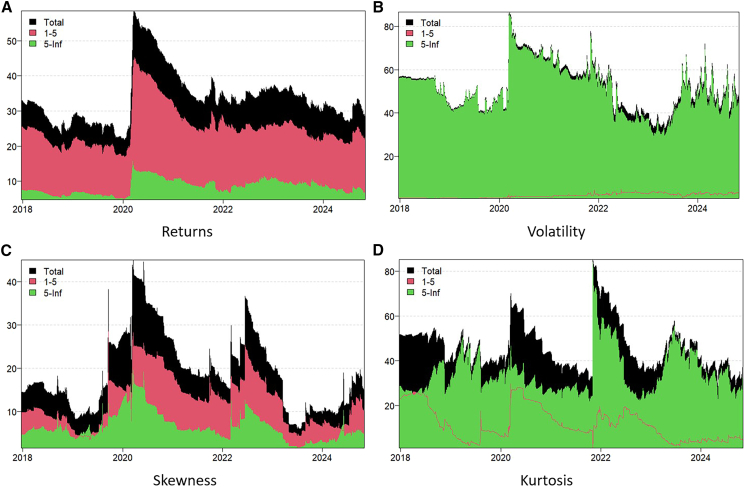
Dynamic overall spillovers (A) Return spillover effect, (B) Volatility spillover effect, (C) Skewness spillover effect, (D) Kurtosis spillover effect.

Static spillover effects indicate the intensity and direction of markets but do not explain the dynamic process associated with the spillover. Figure 3 shows the dynamic spillover effects of returns, volatility, skewness, and kurtosis in the carbon, energy, and AI markets. The black shaded area represents the total spillover effect; the red and green shaded areas represent the short-term and the long-term spillover effects, respectively. A larger shaded area indicates there is a higher spillover effect among the markets.

The results show that the spillover effects of all orders have significant time-varying characteristics. The spillovers of volatility and kurtosis are higher overall compared with those of returns and skewness, fluctuating between 20% and 85%. The red region dominates the return and skewness spillovers; the green region dominates the volatility and kurtosis spillovers. This indicates that return and skewness are dominated by short-term spillovers and volatility and kurtosis are dominated by long-term spillovers. These outcomes are consistent with the findings of the static spillovers.

In addition, there are multiple distinct peaks and periods of turbulence on the spillover curves. These may be because spillovers between markets are affected by external shocks, including unexpected events, economic events, and GPRs.^61^ For example, during the COVID-19 pandemic in 2020 and the outbreak of the Russia-Ukraine war in 2022, there was clear volatility in global markets, and the spillover effect reached a high level.

### Brock-Dechert-Scheinkman nonlinearity test

This section analyzes the factors influencing spillover effects from macro and micro perspectives. Table 11 shows the descriptive statistics of the variables. The Augmented Dickey-Fuller test is tested for each variable, and all variables pass the smoothness test after first-order differencing. In addition, this study conducts the Brock-Dechert-Scheinkman (BDS) nonlinearity test.^62^ Table 12 shows the results. All variables are nonlinear over the study period. This affirms the applicability of the MQQR method for this study.

**Table 11:** Descriptive statistics

	N	Mean	Std. Dev	Median	Min	Max	Skewness	Kurtosis	ADF
TCI1	1748	0.00	0.45	−0.05	−2.45	6.08	4.72	47.26	−8.61∗∗∗
TCI2	1748	0.00	1.84	−0.01	−10.84	30.53	3.63	58.62	−13.44∗∗∗
TCI3	1748	0.00	1.01	0.00	−5.72	22.21	12.11	243.02	−13.07∗∗∗
TCI4	1748	0.01	1.87	0.10	−10.81	52.36	14.94	390.35	−12.13∗∗∗
GPR	1748	4.68	0.46	4.70	2.25	6.29	−0.26	0.95	−5.89∗∗∗
GEPU	1748	5.49	0.22	5.46	4.83	6.07	−0.05	0.04	−3.62∗∗
CR	1748	3.81	0.25	3.83	3.04	4.50	0.09	0.96	−8.30∗∗∗
AITP	1748	3.87	0.82	4.15	1.15	5.40	−0.87	−0.02	−4.72∗∗∗
INVA	1748	3.07	0.47	2.94	1.54	4.30	0.19	−0.52	−5.95∗∗∗

^∗∗∗^, ^∗∗^, and ^∗^ denote 1%, 5%, and 10% level of significance, respectively.

**Table 12:** The results of the BDS test

	TCI1	TCI2	TCI3	TCI4	GPR	GEPU	CR	AITP	INVAI
2.dim.z-sat	9.095	19.411	14.947	12.024	28.766	349.802	250.961	166.486	163.500
Prob	0.00	0.00	0.00	0.00	0.00	0.00	0.00	0.00	0.00
3.dim.z-sat	9.831	25.151	15.977	13.496	32.718	690.693	452.490	270.638	255.438
Prob	0.00	0.00	0.00	0.00	0.00	0.00	0.00	0.00	0.00

### MQQR results and discussion

**Figure 4:**
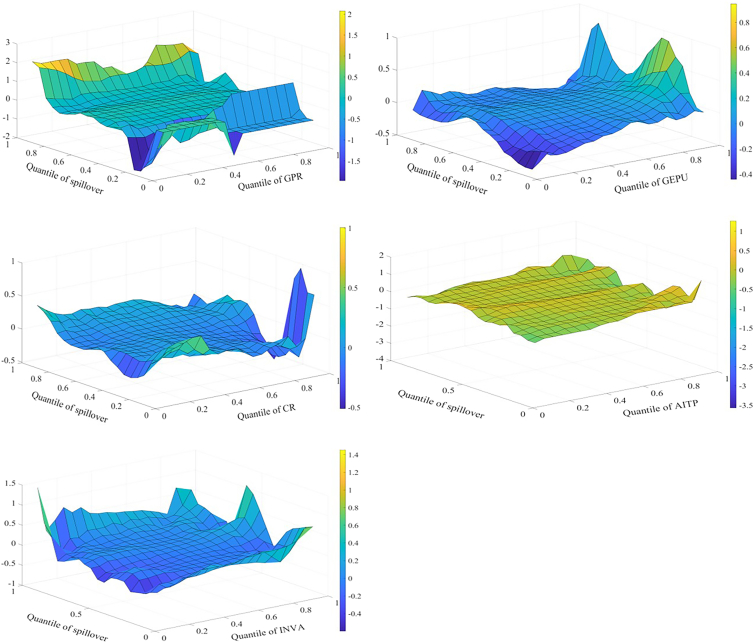
Factors influencing returns spillover

Spillover effects across markets are affected by multiple influencing factors that interact with one another. Therefore, this study employs the MQQR model to comprehensively examine the various factors affecting spillover effects. Figure 4 shows the effects of each factor on the spillover effect for returns. First, the situation where influencing factors are at different quantiles is analyzed. When GEPU and INVA are in the higher quantile (0.8–0.95), both have a significant positive impact on return spillovers. Economic policy is an important driver of credit flows and financial cycles; uncertainty increases the market’s sensitivity to negative information and affects investor expectations. Investors subsequently adjust their portfolios and change asset allocations. As a result, EPU affects supply and demand, increasing spillovers between markets. When investors pay more attention to the three markets of interest, market sentiment spreads quickly through channels such as social media. This can trigger a herd effect. As a result, investors increase the frequency of adjustments to assets. This increases the spillover effect among markets.

When spillover effects are in different quantiles, the impact of influencing factors on the spillover effect also varies. AITP mainly reduces return spillovers. AI technology can improve market efficiency and reduce arbitrage opportunities through real-time data analytics, potentially reducing risk transmission between markets. Further, AI technology can help investors optimize their portfolios and better diversify risks, reducing market return spillovers. When return spillovers are in the high quantile (0.80–0.95), GPR, CR, and INVA all have a strong positive relationship with return spillovers. The effect of GPR is larger. GPR, CR, and other factors may work together in times of high market stress to affect energy supply and infrastructure across markets, exacerbating intermarket spillovers. During periods of high market uncertainty, investors may pay more attention to risk factors, leading to faster information transmission and more sensitive market reactions to negative information. This may increase return spillovers.

**Figure 5:**
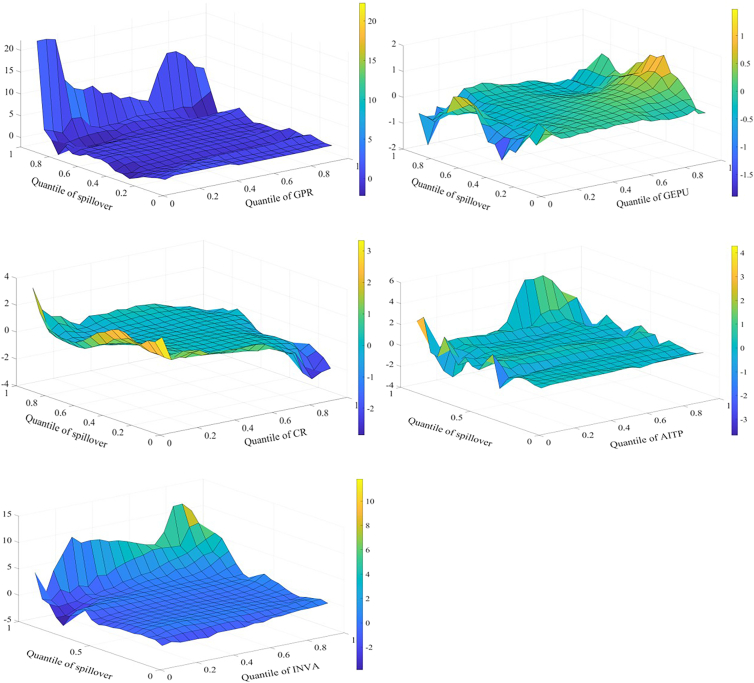
Factors influencing volatility spillover

The effect of each factor on the volatility spillover effect is shown in Figure 5. Similar to the results for return spillovers, when GEPU and INVA are in the higher quantile (0.80–0.95), both have a significant positive impact on volatility spillovers. When the volatility spillover effect is in the high quantile (0.80–0.95), GPR and INVA are positively related to the effect. In addition, the results indicate that CR increases volatility spillovers when CR is in the low quantile and suppresses volatility spillovers when CR is in the high quantile. There are some possible reasons for this. During periods of low CR, short-term factors such as policy deregulation and technological breakthroughs may increase volatility spillovers between markets. When CR is high, markets are warned promptly of crisis events, such as extreme weather events. This allows investors to adjust their long-term positions and reduce their response to short-term volatility. Similar to the results of return spillover, when volatility spillovers are in the high quantile (0.80–0.95), GPR, CR, and INVA all have a strong positive relationship with volatility spillovers.

**Figure 6:**
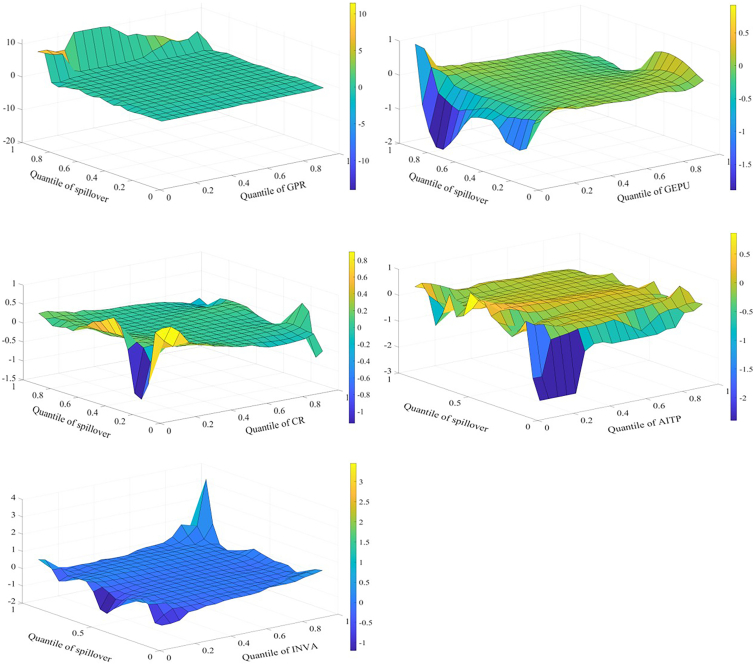
Factors influencing skewness spillover

Figure 6 shows the effect of each factor on the skewness spillover effect. When GEPU is in the low quantile (0.05–0.25), it lowers the effect. When economic policies are more stable, investors have more consistent expectations about future policies, possibly leading to lower inter-market risk spillovers. Further, during periods of economic policy stability, there are fewer arbitrage opportunities, and investors may focus more on factors specific to their respective markets than on changes in macro policy. This may lower skewness spillovers. When CR is in the low quantile (0.05–0.25), it increases skewness spillovers. When the skewness spillover effect is in the higher quantile (0.80–0.95), GPR mainly increases the skewness spillover effect. This is consistent with the previous analysis.

**Figure 7:**
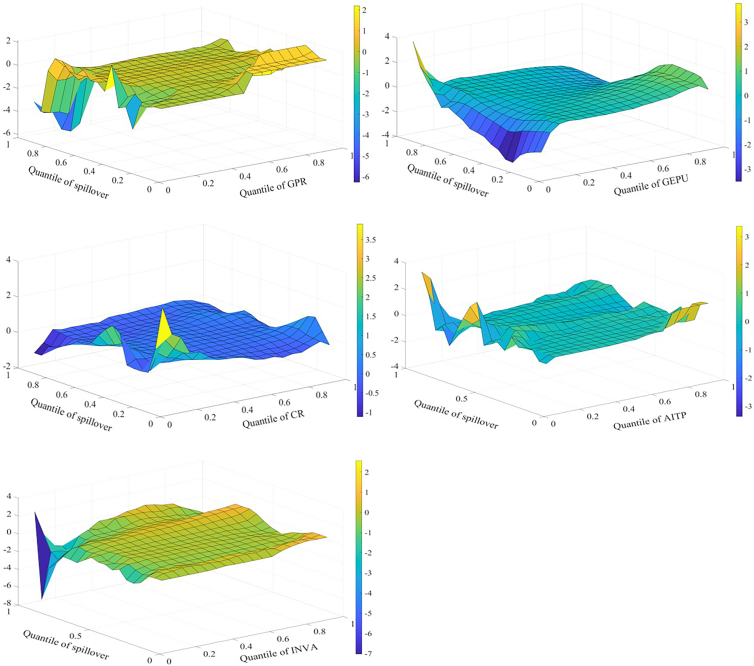
Factors influencing kurtosis spillover

Figure 7 shows the effects of the factors on kurtosis spillovers. When CR is in the lower quantile, it mainly contributes to these spillovers, consistent with the previous findings. When INVA is in the lower quantile, it slows down the kurtosis spillovers. The possible reasons are as follows. First, when investors are paying less attention, there is inefficient inter-market information transmission and a lack of common information or trading behavior among markets. Second, low investor attention may lead to reduced participation in the market. This lowers the transmission effect of volatility extremes between markets.

When kurtosis spillovers are in the high quantile (0.85–0.95), GPR and AITP can lower kurtosis spillovers. This result differs from the previous findings. This may be because when kurtosis spillovers are strong and GPR is elevated, investors may adopt hedging strategies and reduce their investments. This may lower the linkage of extreme events. In addition, advances in AI technology, such as predictive algorithms and risk modeling, increase the ability of markets to anticipate and respond to extreme events. This may reduce the linkage of extreme events.

It is found that there are several common conclusions. When GEPU and INVA are in the higher quantile (0.8–0.95), both have a significant positive impact on return and volatility spillover effects. Among the return, volatility, and skewness spillover effects, when the spillover effects are in the higher quantile range (0.8–0.95), GPR, CR, and INVA all exert a significant positive influence on the spillover effect. This indicates that GPR, GEPU, CR, and INVA primarily amplify spillover effects across markets. Among all spillover effects, AITP predominantly depresses spillover effects. This demonstrates that technological progress plays a significant role in mitigating risk spillovers between markets.

### Robustness tests

**Figure 8:**
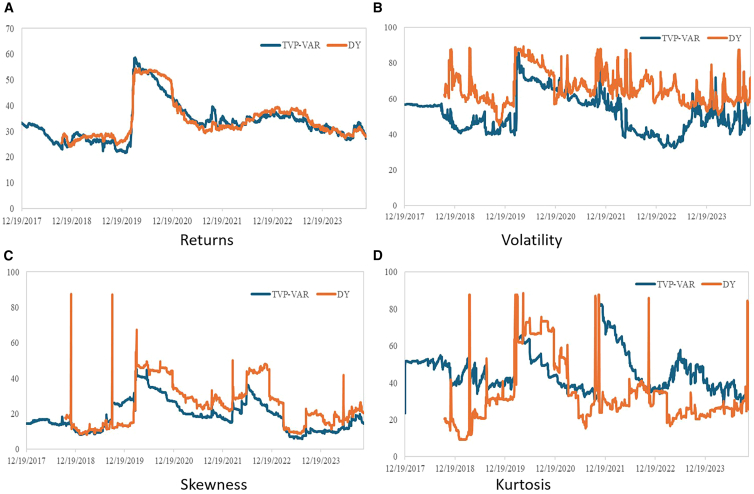
Dynamic overall spillovers (A) Return spillover effect, (B) Volatility spillover effect, (C) Skewness spillover effect, (D) Kurtosis spillover effect.

**Figure 9:**
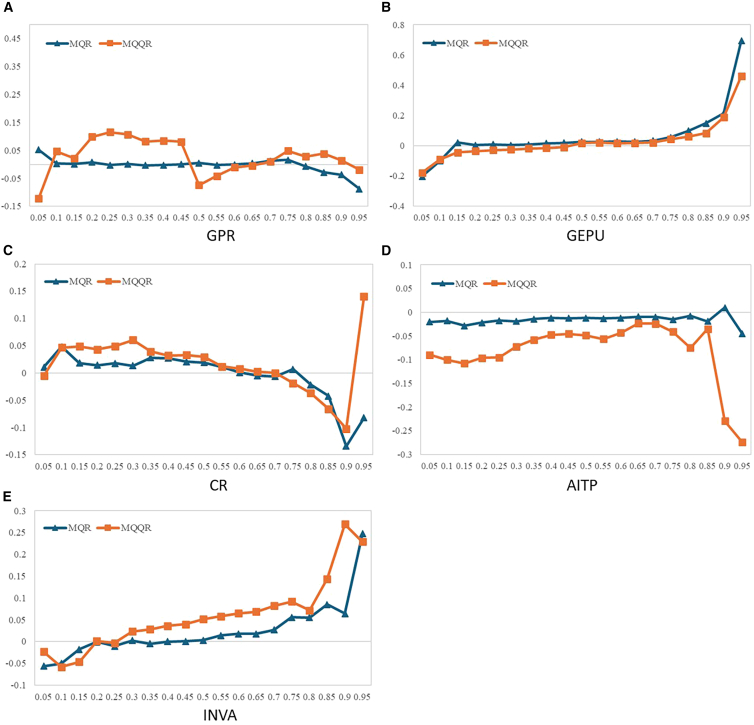
Comparison of the MQQR and MQR (A) The impact of GPR. (B) The impact of GEPU. (C) The impact of CR. (D) The impact of AITP. (E) The impact of INVA.

To conduct the robustness tests, this study uses the connectedness model proposed by Diebold and Yilmaz (2012)^63^ and Diebold and Yılmaz (2014)^64^ in place of the TVP-VAR model. Table 13 shows that the results remain consistent for return, volatility, skewness, and kurtosis spillover indices among carbon, energy, and AI markets when using different estimation methods. The rolling window method is used to analyze the robustness of the dynamic spillover effect. Figure 8 shows that the trends with the two methods are consistent, indicating the results are robust. To assess the robustness of the influencing factors, this study applies the approach of Alola et al. (2023)^65^ and compares the results of the multivariate quantile regression model (MQR) with the results of the average of the MQQR. The results in Figure 9 show that the trends estimated by MQQR and MQR are approximately the same. This confirms the validity and robustness of the results.

**Table 13:** Total spillover results (Diebold and Yilmaz model)

	AI	EUA	Gas	Oil	Coal	Solar	Wind	Nuclear	FROM
**Panel A: Return**									

AI	43.48906	1.163964	0.295325	2.539081	0.12343	19.72323	18.02091	14.645	7.063868
EUA	2.322124	86.04362	0.487384	2.939502	1.897408	1.007142	2.616796	2.686028	1.744548
Gas	0.622004	0.492048	94.96258	0.985678	0.775273	0.817532	0.230078	1.11481	0.629678
Oil	4.655893	2.630055	0.964818	77.39761	1.299012	3.647672	3.784353	5.620586	2.825299
Coal	0.113734	1.102947	0.722619	1.789037	95.81342	0.101072	0.111683	0.245491	0.523323
Solar	20.56195	0.517844	0.585161	2.137865	0.043184	46.02747	20.1303	9.996224	6.746567
Wind	18.37305	1.284515	0.194191	2.087324	0.093599	19.6775	41.97536	16.31446	7.253079
Nuclear	16.49763	1.491865	0.581068	3.527381	0.017409	10.66403	18.32373	48.8969	6.387888
TO	7.893298	1.085405	0.478821	2.000733	0.531164	6.954772	7.902231	6.327825	33.17425

**Panel B: Volatility**									

AI	29.71597	0.173416	0.041904	15.91193	0.420865	17.53132	17.88426	18.32032	8.785503
EUA	15.18458	42.1868	0.040049	6.551987	3.692901	10.50168	12.79795	9.044055	7.22665
Gas	7.338028	0.205109	74.87754	0.272904	6.350997	1.94215	3.14051	5.872759	3.140307
Oil	18.49215	0.767082	0.330288	41.99383	1.039508	14.85065	9.556003	12.97048	7.250771
Coal	0.114515	7.457042	5.052307	0.500177	83.38181	0.4677	2.551305	0.47515	2.077274
Solar	21.05667	0.412146	0.024921	15.5657	0.107095	27.84616	19.3806	15.6067	9.019229
Wind	22.3099	0.350372	0.101294	16.95065	0.185737	18.44564	22.13273	19.52368	9.733408
Nuclear	23.65597	0.650098	0.072476	16.4455	0.086514	16.35879	17.57643	25.15421	9.355723
TO	13.51898	1.251908	0.707905	9.024857	1.485452	10.01224	10.36088	10.22664	56.58887

**Panel C: Skewness**									

AI	77.78838	0.007595	0.040603	0.017806	0.269089	10.14562	6.529713	5.201193	2.776452
EUA	0.242363	92.69563	0.190148	0.040428	2.90315	1.281131	1.126845	1.520309	0.913047
Gas	1.045676	0.231131	96.66605	0.010429	0.24624	1.0656	0.218305	0.516573	0.416744
Oil	0.030522	0.291128	0.010403	98.85946	0.643527	0.049612	0.026066	0.089283	0.142568
Coal	0.03346	0.334536	0.17676	0.097431	98.98783	0.069245	0.038888	0.261851	0.126521
Solar	8.872748	0.112494	0.069799	0.045055	0.021028	73.77591	11.80335	5.299616	3.278012
Wind	6.022995	0.270307	0.182041	0.023589	0.028575	17.06122	68.53635	7.874917	3.932956
Nuclear	5.024013	0.098367	0.245392	0.041555	0.031377	8.836117	8.430025	77.29315	2.838356
TO	2.658972	0.168195	0.114393	0.034537	0.517873	4.813569	3.521649	2.595468	14.42466

**Panel D: Kurtosis**									

AI	64.67143	0.025007	0.086507	2.325262	0.005196	16.70054	7.210645	8.975415	4.416071
EUA	0.738972	88.26377	0.184851	1.158153	0.010168	4.205626	2.021978	3.416481	1.467029
Gas	5.408888	0.087488	91.50781	0.415881	0.010776	2.024278	0.082438	0.462446	1.061524
Oil	2.036317	0.014667	0.117729	81.35508	0.002191	10.47628	0.975449	5.022282	2.330615
Coal	0.01208	0.006455	0.00646	0.004001	99.93774	0.016258	0.010898	0.006106	0.007782
Solar	14.31002	0.047792	0.05012	11.09326	0.007205	61.65986	3.641513	9.19024	4.792518
Wind	8.341612	0.056607	0.13891	3.251897	0.281927	12.19391	68.62559	7.109547	3.921801
Nuclear	8.468332	0.165698	0.182977	7.880193	0.003397	21.74929	5.558271	55.99185	5.501019
TO	4.914527	0.050464	0.095944	3.26608	0.040108	8.420772	2.437649	4.272815	23.49836

## Discussion

This study examines several variables, including returns, volatility, skewness, and kurtosis, to analyze the spillover effects in different time and frequency domains in the AI, carbon, and energy markets. The TVP-VAR model is applied first, followed by a multivariate MQQR model, to analyze the multiple influencing factors. The study combines macro and micro perspectives to explore the impact of GRP, GEPU, CR, AITP, and INVA on spillover effects. It also analyzes whether there are differences in the degree of impact on spillover effects in different quantile levels.

In terms of market spillovers, there are three key results. First, there are strong spillovers between carbon, energy, and AI markets. These occur at the level of returns and volatility and at the level of skewness and kurtosis. Spillovers of volatility and kurtosis exceed those of returns and skewness as a whole. Second, the static spillover results show that the AI and new energy markets are the main risk-transmitting markets with respect to return and volatility spillovers; for skewness and kurtosis spillovers, the spillover effect is increased for conventional energy. At all orders of moments, the carbon market is consistently a net risk receiver. Return and skewness connectedness between markets are more affected by short-term effects, and volatility and kurtosis connectedness are more affected by long-term effects. Third, the dynamic spillover results indicate that spillovers across order moments in the carbon, energy, and AI markets are significantly time-varying. Major crisis events tend to significantly increase market spillover effects.

In terms of spillover drivers, the impact of each factor on spillover effects varies across quantiles. Specifically, GPR positively affects spillovers in returns, volatility, and skewness when spillovers are in the higher quantiles. GEPU contributes to spillovers in returns, volatility, and kurtosis when GEPU is in the higher quantiles but suppresses market skewness spillovers in the lower quantiles. CR mainly contributes to spillovers at lower quantiles and suppresses spillovers at higher quantiles. AITP has mostly negative effects on spillover effects; this impact shifts to positive effects when both volatility spillovers and technological progress are in the higher quantiles. At the micro level, INVA mainly contributes to return, volatility, and skewness spillovers when investor attention and market spillovers are in the high quantiles.

These findings are important for investors to optimize investment portfolios. First, investors should establish a comprehensive monitoring system, instead of viewing individual markets in isolation. When constructing market investment portfolios and hedging strategies, investors should focus on the risk spillover effects of higher-order moments across markets, formally incorporating tail risks (skewness) and “black swan” risks (kurtosis) into their risk assessment frameworks. Second, risk-averse investors should be particularly cautious about traditional energy, as they are the primary transmitter of extreme risks. Investors should focus on trends in AI and new energy markets, as the AI and new energy markets are the primary risk transmission markets in terms of return and volatility spillover effects. Third, the carbon market is a consistent net risk recipient, so investors can use it as a risk hedging tool. However, they should remember the potential risks posed by the carbon market’s instability and allocate their investments appropriately.

The findings also offer some useful information for policymakers in improving risk management systems. First, the policymakers should develop separate long-term and short-term policy tools to increase risk management efficiency when addressing market spillover effects. Second, dynamic monitoring and emergency response mechanisms are needed to assess geopolitical changes and economic policy changes. Policymakers should pay close attention to changes in the global geopolitical situation and flexibly adopt diversified portfolios to minimize risks. Emergency response planning should be strengthened to address the risks of underestimating the impacts of climate change. Finally, as technological innovations continue in the AI field, policymakers should promptly address the coordination between technological innovations and market dynamics.

### Limitations of the study

This study offers valuable insights for decision-making, yet several limitations remain. First, although daily closing prices reveal inter-market linkages, they overlook within-day dynamics and high-frequency interactions. Second, the analysis focuses only on AI, carbon, and energy markets, without considering risk transmissions through other interconnected markets, which may simplify the actual risk network. Third, spillover determinants incorporate only extreme-weather-based CR and investor attention as micro-level factors. Finally, the MQQR model uncovers statistical associations rather than establishing strict causal relationships, limiting causal interpretation of spillover drivers.

These limitations also highlight directions for future research. First, using higher-frequency data, such as minute-level prices, could capture finer spillover dynamics. Second, future work could build more complex and interconnected market network models to reveal comprehensive transmission pathways, including intermediaries beyond the focal markets. Third, spillover determinants could integrate both physical and transition CRs, alongside richer micro-level behavioral factors. Finally, methodological enhancements, such as structured causal models or instrumental-variable approaches, could strengthen causal identification in spillover analysis.

## Resource availability

### Lead contact

Further information and requests for resources should be directed to and will be fulfilled by the lead contact, Bin Su (subin@nus.edu.sg).

### Materials availability

This study did not generate new unique reagents.

### Data and code availability

• The descriptions of data are listed in the key resources table.
• The codes written in R language are available upon request from the lead contact.
• Any additional information required to re-analyze the data reported in this paper are available from the lead contact upon request.

## Acknowledgments

The authors gratefully acknowledge the financial support provided by the 10.13039/100014718National Natural Science Foundation of China (nos.72274215), 10.13039/100017414Shandong Provincial Social Science Foundation (25BLJJ13), and 10.13039/100007219Shandong Provincial Natural Science Foundation (no. ZR2025MS1164).

## Author contributions

Conceptualization: M.Z. and B.S.; methodology: M.Z. and Y.P.; data curation: M.Z.; formal analysis: M.Z. and D.Z.; writing-original draft: M.Z. and Y.P.; writing-review and editing: M.Z., Y.P., B.S., and D.Z.; validation: B.S; funding acquisition: M.Z.

## Declaration of interests

The authors declare no competing interests.

## STAR★Methods

### Key resources table

**Table:** 

REAGENT or RESOURCE	SOURCE	IDENTIFIER
**Deposited data**		

European Union allowance futures	Intercontinental Exchange	https://www.investing.com/
Rotterdam coal futures	Intercontinental Exchange	https://www.investing.com/
Brent crude oil futures	Intercontinental Exchange	https://www.investing.com/
Port Henry natural gas futures	Energy Information Administration	https://www.eia.gov/
NASDAQ OMX Solar Index	NASDAQ website	https://indexes.nasdaqomx.com/
NASDAQ OMX Wind Index	NASDAQ website	https://indexes.nasdaqomx.com/
MVIS® Global Uranium and Nuclear Index	Marketventor website	https://www.marketvector.com/
NASDAQ Global CTA Artificial Intelligence and Robotics Index	NASDAQ website	https://indexes.nasdaqomx.com/
Geopolitical risk index	Caldara and Iacoviello (2022)^66^	Caldara and Iacoviello (2022)^66^
Global economic policy uncertainty index	Baker et al. (2016)^67^	http://www.policyuncertainty.com/
Global frequency of climate-related disasters	Emergency Events Database	https://public.emdat.be/
AI patents	World Intellectual Property Organization	https://www.oecd.org/
Investor attention index	Google Trends	https://trends.google.com/

**Software and algorithms**		

R 4.4.2	The R Project for statistical computing	https://www.r-project.org/
MATLAB R2023a	MATLAB	https://www.mathworks.com/

### Experimental model and study participant details

There are no experimental model or study participants to include in this study.

### Method details

#### Data for carbon, energy, and AI markets

The markets data sources for this study are summarized in Table 12. First, the carbon market is represented by European Union allowance futures (EUA). The European Union Emissions Trading System is the world’s first large-scale carbon emissions trading market, and accounts for 87% of the global carbon market share. As such, the EUA effectively represents development trends in the global carbon market.^27^^,^^28^ Second, the development of the AI market is represented by the NASDAQ Global CTA Artificial Intelligence and Robotics Index. This equity index focuses on the AI and robotics sector, and is designed to track the aggregate performance of companies in related fields.^36^

Third, this study divides the energy market into traditional energy markets and new energy markets. Traditional energy markets include coal (measured by the Rotterdam coal futures price), oil (measured by the Brent crude oil futures price), and natural gas (measured by the Port Henry natural gas futures price).^28^^,^^29^ New energy markets include solar (as measured by the NASDAQ OMX Solar Index), wind (as measured by the NASDAQ OMX Wind Index), and nuclear (as measured by the MVIS Global Uranium and Nuclear Index).^24^

The data for the AI, solar, and wind markets are from the official NASDAQ website; the data for the natural gas market are from the Energy Information Administration (EIA); and the data for the nuclear energy market are from the Marketventor. The data for the carbon, coal, and oil markets are from the Intercontinental Exchange.

Market changes over a short period of time are better captured by daily data than weekly-frequency and monthly-frequency data. This study uses daily data from December 18, 2017 to October 31, 2024. The prices of financial assets are generally non-stationary due to the relatively large magnitude of changes; however, the return series are usually smooth and have good statistical characteristics.^51^^,^^68^^,^^69^ Therefore, this study processes the initial data and then uses the logarithmic rate of return on asset prices. The logarithmic rate of return is calculated as follows:

(Equation 1) $$r_{i,t}=100\times \ln\left(\frac{P_{i,t}}{P_{i,t-1}}\right)=100\times \left(\ln\;P_{i,t}-\ln\;P_{i,t-1}\right)$$

where *r*_*i*,*t*_ represents the return of index *i* on day *t*. The term *P*_*i*,*t*_ and *P*_*i*,*t*-1_ denote the closing price of index *i* on day *t* and *t*-1, respectively.

#### Data for influencing factors

This study explores the factors influencing risk spillover effects from a macro-environmental perspective and a micro-investor risk perception perspective. For the dependent variable, risk spillover effects are measured using a daily total spillover index of returns, volatility, skewness, and kurtosis. For the independent variables, at a macro perspective, this study analyzes the impact of political, economic, social, and technological factors on spillover effects using the PEST (Political-Economic-Social-Technological) analytical model.

Table 13 summarizes the data for influencing factors. In this model, GPR represents political factors and is measured using the geopolitical risk index compiled by Caldara and Iacoviello (2022).^66^ GEPU represents economic factors and is measured using the global economic policy uncertainty index measured by Baker et al. (2016).^67^ CR represents social factors and is measured using the global frequency of climate-related disasters, with data from the Emergency Events Database. AITP represents technological factors^16^^,^^47^; the study applies Yang (2022)^70^ and measures AITP by the number of AI patents, with data from the World Intellectual Property Organization. The number of patents about AI published on each date is manually counted, and the data are processed using the seven-day moving average method.

At a micro perspective, this study analyzes the impact of the Google Search Volume Index,^53^ with data from Google Trends. This study draws from Chronopoulos et al. (2018)^71^ for daily frequency data of carbon, energy, and AI market concerns. These are weighted to obtain the investor attention index.

#### GJRSK model

The time series data across the three examined markets have non-normal distributional properties, showing distinct leptokurtic features with heavy-tailed characteristics and pronounced leverage effects. To measure the conditional volatility, skewness, and kurtosis of the data for the carbon, energy, and AI markets, this study applies the GJRSK model proposed by Nakagawa and Uchiyama (2020)^72^ to build a higher-order moment model. The model is based on the Glosten-Jagannathan-Runkle GARCH model, with asymmetric response to positive and negative shocks. The GJRSK model is specified as follows:

(Equation 2) $${\text{r}}_{\text{t}}=a_{1}{\text{r}}_{\text{t}-1}+\varepsilon_{t}$$

(Equation 3) $$h_{t}=\beta_{0}+\beta_{1}\varepsilon_{t-1}^{2}+\beta_{2}h_{t-1}+\beta_{3}\varepsilon_{t-1}^{2}I_{\left\{\eta_{t-1}<0\right\}}$$

(Equation 4) $$s_{t}=\gamma_{0}+\gamma_{1}\eta_{t-1}^{3}+\gamma_{2}s_{t-1}+\gamma_{3}\eta_{t-1}^{3}I_{\left\{\eta_{t-1}<0\right\}}$$

(Equation 5) $$k_{t}=\delta_{0}+\delta_{1}\eta_{t-1}^{4}+\delta_{2}k_{t-1}+\delta_{3}\eta_{t-1}^{4}I_{\left\{\eta_{t-1}<0\right\}}$$

(Equation 6) $$\eta_{t}=h_{t}^{-1/2}\varepsilon_{t}$$

(Equation 7) $$\eta_{t}|{\text{I}}_{\text{t}-1}∼g\left(0,1,{\text{s}}_{\text{t}},{\text{k}}_{\text{t}}\right)$$

where *a*_1_ is a parameter of the autoregressive model and *β*_*i*_,*γ*_*i*_,*δ*_*i*_ are parameters of the GJRSK model, where *i* = 0,1,2,3. The term *I*_*A*_ is an indicator function with *I*_*A*_ = 1 if A is true and *I*_*A*_ = 0 if A is false; $I_{\left\{\eta_{t-1}<0\right\}}=1$ when *η*_*t*-1_ < 0, otherwise $I_{\left\{\eta_{t-1}<0\right\}}=0$. Thus, the positive disturbance of information on market volatility is *β*_1_, and the negative disturbance is *β*_1_+*β*_3_, where *β*_3_ is the leverage effect function. The term *g* is a probability density function with a mean of 0, variance of 1, skewness of *s*_*t*_, and kurtosis of *k*_*t*_. The rates of return on the carbon, energy, and AI markets are *r*_*t*_, computed by 100×(*lnP*_*t*_-*lnP*_*t*-1_), where *P*_t_ is the daily market closing price. The individual parameters of the GJRSK model are estimated by maximizing the likelihood function.

#### TVP-VAR model

Based on the above dataset, this study employs the TVP-VAR model to analyze spillover effects across markets. The TVP-VAR model extends the connectivity model of Diebold and Yilmaz (2012)^63^ and is commonly used to study the time-varying characteristics of volatility spillovers among different markets. The model flexibly describes the interrelationships between variables by introducing time-varying parameters. The model does not need to set the rolling window size independently. This avoids the subjective setting of window size and loss of data, and has the benefits of being insensitive to outliers and allowing a time-varying variance-covariance structure. This study distinguishes short-term and long-term spillover effects. The basic model of TVP-VAR (*p*) is as follows.

(Equation 8) $${\text{x}}_{\text{t}}=\Phi_{1\text{t}}{\text{x}}_{\text{t}-1}+\Phi_{2\text{t}}{\text{x}}_{\text{t}-2}+\ldots +\Phi_{\text{pt}}{\text{x}}_{\text{t}-p}+\xi_{t},\xi_{t}∼N\left(0,\Sigma_{t}\right)$$

where *x*_*t*_ and *ξ*_*t*_ are N × 1 dimensional vectors; Φ_*it*_ denotes the time-varying coefficient matrix of the N × N dimensional vector autoregressive model; and Σ_*t*_ denotes the N × N dimensional time-varying variance-covariance matrix.

Using the (N × N) matrix lag-polynomial Φ(*L*), the model is rewritten into a TVP-VMA form, where *I*_*N*_ is the unit matrix:

(Equation 9) $$x\left(t\right)=\Psi \left(L\right)\xi_{t}={\left[\Phi \left(L\right)\right]}^{-1}\xi_{t}$$

(Equation 10) $$\Phi \left(L\right)=\left[{\text{I}}_{\text{N}}-\Phi_{1\text{t}}\text{L}-\ldots -\Phi_{\text{p}\text{t}}{\text{L}}^{\text{p}}\right]$$

Then, the generalized forecast error variance decomposition (GFEVD) model is computed to obtain the following equations.^73^^,^^74^ This represents the effect of a shock to market *j* on market *i*, and relates to its forecast error variance:

(Equation 11) $$\theta_{ijt}\left(H\right)=\frac{{\left(\Sigma_{t}\right)}_{jj}^{-1}\sum_{h=0}^{H}{\left({\left(\Psi_{h}\Sigma_{t}\right)}_{ijt}\right)}^{2}}{\sum_{h=0}^{H}{\left(\Psi_{h}\Sigma_{t}\Psi_{h}^{\prime }\right)}_{ii}}$$

(Equation 12) $${\tilde{\theta }}_{ijt}\left(H\right)=\frac{\theta_{ijt}\left(H\right)}{\sum_{k=1}^{N}\theta_{ijt}\left(H\right)}$$

(Equation 13) $$\sum_{i=1}^{N}{\tilde{\theta }}_{ijt}\left(H\right)=1$$

(Equation 14) $$\sum_{i=1}^{N}\sum_{j=1}^{N}{\tilde{\theta }}_{ijt}\left(H\right)=N$$

where *θ*_*ijt*_(*H*) is normalized to obtain ${\tilde{\theta }}_{ijt}\left(H\right)$, and ${\tilde{\theta }}_{ijt}\left(H\right)$ measures the contribution of market *j* to the variance of the forecast error of market *i* at horizon *H*.

This leads to the generation of an expression for the connectedness measure, including directional total connectedness *TO*_*it*_(*H*) and *FROM*_*it*_(*H*), directional net connectedness *NET*_*it*_(*H*), and directional network pairwise connectedness *NPDC*_*ijt*_(*H*):

(Equation 15) $$TO_{it}\left(H\right)=\sum_{i=1,i\neq j}^{N}{\tilde{\theta }}_{jit}\left(H\right)$$

(Equation 16) $$FROM_{it}\left(H\right)=\sum_{j=1,i\neq j}^{N}{\tilde{\theta }}_{ijt}\left(H\right)$$

(Equation 17) $${\text{NET}}_{\text{it}}\left(H\right)={\text{TO}}_{\text{it}}\left(H\right)-{\text{FROM}}_{\text{it}}\left(H\right)$$

(Equation 18) $$NPDC_{ijt}\left(H\right)={\tilde{\theta }}_{ijt}\left(H\right)-{\tilde{\theta }}_{jit}\left(H\right)$$

where *TO*_*it*_(*H*) denotes the level of spillovers from variable *i* to all variables *j*, and *FROM*_*it*_(*H*) denotes spillovers received by variable *i* from all variables *j*. *NET*_*it*_(*H*) denotes directional net connectedness. If *NET*_*it*_(*H*) is larger than 0, then variable *i* is a shock transmitter; otherwise, it is a shock receiver. The term *NPDC*_*ijt*_(*H*) denotes directional network pairwise connectedness. If *NPDC*_*ijt*_(*H*) > 0 (or *NPDC*_*ijt*_(*H*) < 0), the effect of variable *j* on variable *i* is more (or less) than the effect that variable *i* has on variable *j*.

The modified total connectedness index proposed by Chatziantoniou and Gabauer (2021)^75^ and Gabauer (2021)^76^ is further defined to measure the degree of total connectedness:

(Equation 19) $$TCI_{t}\left(H\right)=\frac{N}{N-1}\sum_{i=1}^{N}TO_{it}\left(H\right)=\frac{N}{N-1}\sum_{i=1}^{N}FROM_{it}\left(H\right)$$

(Equation 20) $$0\leq {\text{TCI}}_{\text{t}}\left(H\right)\leq 1,\text{if}\;\text{H}\to \infty$$

The above equations show the connectedness measures in the time domain. The spectral decomposition method of Stiassny (1996)^77^ is applied to further explore connectedness in the frequency domain. The frequency response function is as follows:

(Equation 21) $$\psi \left(e^{-i\omega }\right)=\sum_{h=0}^{\infty }e^{-i\omega h}\psi_{h},i=\sqrt{-1}$$

The spectral density of *x*_*t*_ at frequency *ω* is then defined as the Fourier transform of the time-varying parameter vector moving average model:

(Equation 22) $$S_{x}\left(\omega \right)=\sum_{h=-\infty }^{\infty }E\left(x_{t}x_{t-h}^{\prime }\right)e^{-i\omega h}=\psi \left(e^{-i\omega h}\right)\Sigma_{t}\psi^{\prime }\left(e^{+i\omega h}\right)$$

#### Combining the spectral density and GFEVD generates the frequency GFEVD

(Equation 23) $$\theta_{ijt}\left(\omega \right)=\frac{{\left(\Sigma_{t}\right)}_{jj}^{-1}{\left|\sum_{h=0}^{\infty }{\left(\Psi \left(e^{-i\omega h}\right)\Sigma_{t}\right)}_{ijt}\right|}^{2}}{\sum_{h=0}^{\infty }{\left(\Psi \left(e^{-i\omega h}\right)\Sigma_{t}\Psi \left(e^{i\omega h}\right)\right)}_{ii}}$$

The frequency GFEVD is further normalized, where ${\tilde{\theta }}_{ijt}\left(\omega \right)$ denotes the percentage of the spectrum of market *i* at the particular frequency *ω* that can be attributed to shocks in market *j*:

(Equation 24) $${\tilde{\theta }}_{ijt}\left(\omega \right)=\frac{\theta_{ijt}\left(\omega \right)}{\sum_{k=1}^{N}\theta_{ijt}\left(\omega \right)}$$

To quantify the connectedness in different frequency bands, all frequencies are collected into a specific range:

(Equation 25) $${\tilde{\theta }}_{ijt}\left(d\right)=\int_{a}^{b}{\tilde{\theta }}_{ijt}\left(\omega \right)d\omega$$

where *d*=(*a*,*b*), *a*∈(-*π*,*π*), *b*∈(-*π*,*π*), *a*<*b*. This study divides the frequency domain into two types of timescales: high frequency (1–5 trading days) and low frequency (>5 trading days). The frequency connectedness measures are as follows:

(Equation 26) $$TO_{it}\left(d\right)=\sum_{i=1,i\neq j}^{N}{\tilde{\theta }}_{jit}\left(d\right)$$

(Equation 27) $$FROM_{it}\left(d\right)=\sum_{i=1,i\neq j}^{N}{\tilde{\theta }}_{ijt}\left(d\right)$$

(Equation 28) $${\text{NET}}_{\text{it}}\left(d\right)={\text{TO}}_{\text{it}}\left(d\right)-{\text{FROM}}_{\text{it}}\left(d\right)$$

(Equation 29) $$NPDC_{ijt}\left(d\right)={\tilde{\theta }}_{ijt}\left(d\right)-{\tilde{\theta }}_{jit}\left(d\right)$$

(Equation 30) $$TCI_{t}\left(d\right)=\frac{N}{N-1}\sum_{i=1}^{N}TO_{it}\left(d\right)=\frac{N}{N-1}\sum_{i=1}^{N}FROM_{it}\left(d\right)$$

where *TO*_*it*_(*d*) measures the total directional connectedness that market *i* transmits to all other markets in a given frequency range; *FROM*_*it*_(*d*) denotes the total directional connectedness that market *i* receives from other markets at a given frequency; *NET*_*it*_(*d*) denotes the net connectedness of market *i*, and *NPDC*_*ijt*_(*d*) denotes the directional net pairwise connectedness that market *j* transmits to market *i*; and *TCI*_*t*_(*d*) denotes the total connectedness of the entire system. (Equation 14), (Equation 15), (Equation 16), (Equation 17) capture the spillover effect under the total time dimension; (Equation 25), (Equation 26), (Equation 27), (Equation 28), (Equation 29) capture the spillover effect at a given frequency.

#### Multivariate quantile-on-quantile regression model

Based on the above dataset, this study employs the MQQR model to analyze the factors influencing spillover effects across markets. The relationship between two variables may change at different quantiles of their respective distributions; to evaluate this, Sim and Zhou (2015)^78^ proposed the quantile-on-quantile regression (QQR) method. QQR extends the traditional quantile regression method and effectively explains how the quantile of the independent variable affects the conditional quantile of the dependent variable. The formula is as follows:

(Equation 31) $$y_{t}=\beta_{0}\left(\theta ,\phi \right)+\beta_{1}\left(\theta ,\phi \right)\left(x_{t}-x^{\phi }\right)+\alpha^{\theta }y_{t-1}+\varepsilon_{t}^{\theta }$$

where *θ* and *ϕ* represent the quantile (0.05–0.95) of the dependent and independent variables, respectively; $\varepsilon_{t}^{\theta }$ is the error term if quantile *θ* is zero. The term *β*_0_ represents the intercept coefficient of the independent variable at quantile *ϕ*, and *β*_1_ represents the effect of quantile *ϕ* of the independent variable on quantile *θ* of the dependent variable.

The QQR is a bivariate model that analyzes the relationship between two variables at different quantiles of their respective distributions. This may omit other important factors. Therefore, Alola et al. (2023)^65^ proposed the MQQR method to include multiple independent variables. The MQQR method evaluates the relationship between the quantile of the dependent variable and any number of quantiles of the independent variables. This enables observations of the effects of the respective variables on the dependent variable at different quantiles. Its formula is as follows:

(Equation 32) $$\begin{array}{l} y_{t}=\beta_{0}\left(\theta ,\phi_{1},\phi_{2}...\phi_{n}\right)+\beta_{1}\left(\theta ,\phi_{1}\right)\left(x_{1t}-x_{1}^{\phi_{1}}\right)+\beta_{2}\left(\theta ,\phi_{2}\right)\left(x_{2t}-x_{2}^{\phi_{2}}\right)+...+\beta_{n}\left(\theta ,\phi_{n}\right)\left(x_{nt}-x_{n}^{\phi_{n}}\right)+\\ \alpha^{\theta }y_{t-1}+\epsilon_{t}^{\theta } \end{array}$$

where *x*_1_,*x*_2_ … *x*_*n*_ is the independent variable; *y* is the dependent variable, where *ϕ*_1_,*ϕ*_2_ … *ϕ*_*n*_ represent the quantile of *x*_1_,*x*_2_ … *x*_*n*_, respectively; and *θ* denotes the quantile of *y*.

### Quantification and statistical analysis

There are no quantification or statistical analyses to include in this paper.
